# Cyclin-dependent kinase 4 is a preclinical target for diet-induced obesity

**DOI:** 10.1172/jci.insight.123000

**Published:** 2018-09-06

**Authors:** Niloy Jafar Iqbal, Zhonglei Lu, Shun Mei Liu, Gary J. Schwartz, Streamson Chua, Liang Zhu

**Affiliations:** 1 Department of Developmental and Molecular Biology and; 2 Department of Medicine, Albert Einstein College of Medicine, New York, New York, USA.

**Keywords:** Metabolism, Obesity

## Abstract

When obesity is caused by consumption of a high-fat diet, the tumor suppressor pRb is phosphoinactivated in the neurons of the mediobasal hypothalamus, a brain area critical for energy-balance regulation. However, the functional relevance of pRb phosphoinactivation in the mediobasal hypothalamus to diet-induced obesity remains unknown. Here, we show that inhibiting pRb phosphorylation in the mediobasal hypothalamus can prevent and treat diet-induced obesity in mice. Expressing an unphosphorylable pRb nonselectively in the mediobasal hypothalamus or conditionally in anorexigenic POMC neurons inhibits diet-induced obesity. Intracerebroventricular delivery of US Food and Drug Administration–approved (FDA-approved) cyclin-dependent kinase 4 (CDK4) inhibitor abemaciclib inhibits pRb phosphorylation in the mediobasal hypothalamus and prevents diet-induced obesity. Oral administration of abemaciclib at doses approved for human use reduces fat mass in diet-induced obese mice by increasing lipid oxidation without significantly reducing lean mass. With analysis of recent literature identifying CDK4 as the most abundantly expressed neuronal CDK in the mediobasal hypothalamus, our work uncovers CDK4 as the major kinase for hypothalamic pRb phosphoinactivation and a highly effective central antiobesity target. As three CDK4/6 inhibitors have recently received FDA approval for life-long breast cancer therapy, our study provides a preclinical basis for their expedient repurposing for obesity management.

## Abstract 2

Inhibiting pRb phosphorylation in the mediobasal hypothalamus can prevent and treat diet-induced obesity in mice.

## Graphical abstract

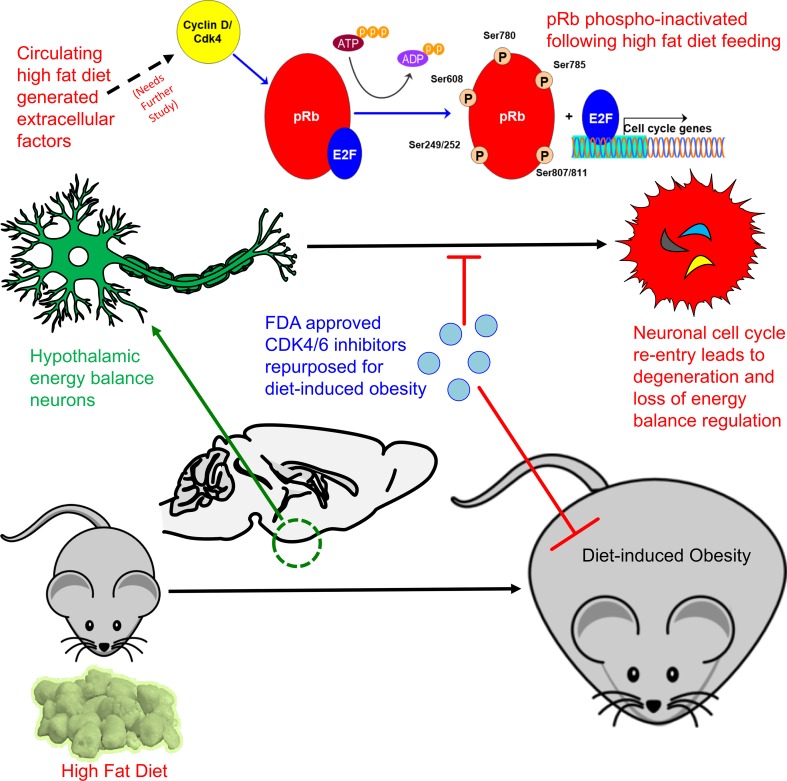

## Introduction

Diet-induced obesity (DIO) is a growing pandemic with significant comorbidities, such as type 2 diabetes and cardiovascular disease. Recent forecasts indicate that 51% of the world population will be obese by 2030 (1), posing major challenges to global health outcomes and costs. Only in rare cases can monogenic etiologies of obesity be identified, such as mutations in genes encoding select hormones (*LEP*, *POMC*) or their receptors (*LEPR*, *MC4R*). The vast majority of obese patients lack such mutations but, instead, have elevated leptin in the circulation, which can be modeled in C57BL/6J mice fed a high-fat diet (HFD, 60%KCal from fat). This suggests that reductions in leptin action can cause DIO. Despite intense research on this topic, elucidating how HFD causes leptin resistance, remains a major challenge in obesity research (2).

Hypothalamus-specific deletion of *LepR* induces the same degree of obesity as *db/db* mice, which globally lack *LepR* function (3). Thus, leptin action deficits in the hypothalamus alone are sufficient to cause obesity, highlighting the importance of hypothalamus neurons in the etiology of obesity. The mediobasal hypothalamus (MBH) comprises the arcuate nucleus (ARC) and the ventromedial nucleus (VMN). The ARC contains major neuronal circuits for energy balance — most importantly, anorexigenic proopiomelanocortin (POMC) neurons and orexigenic agouti-related peptide (AgRP) neurons. It is believed that circulating proinflammatory mediators and other circulating by-products of HFD can directly affect the MBH, with ARC neurons particularly susceptible due to their anatomic proximity to the incomplete blood-brain barrier of the median eminence (4, 5); once present in the ARC, these circulating factors are thought to directly perturb neuron homeostasis, disrupting neuronal postmitotic quiescence and neuronal turnover (6). This further suggests that the direct disruption of ARC energy-balance neurons by HFD may lead to their subsequent leptin insensitivity during an obesogenic state (7). Interestingly, recent studies on the effects of HFD on MBH neurogenesis reached contradictory conclusions, showing that HFD decreased (8) or increased (9) neurogenesis in the MBH and that inhibiting MBH neurogenesis promoted (8) or inhibited (9) DIO. Molecular mechanisms by which HFD can directly affect neuron homeostasis have been postulated, such as activation of the IKKβ/NF-kβ inflammatory pathway (10) or the ER stress responses (11). However, a druggable pathway for correcting MBH neuron homeostasis in order to treat DIO remains elusive.

The tumor suppressor pRb binds and represses the E2F family transcription factors, acting as a master regulator of the G_1_ to M checkpoint during the cell cycle. In adult neurons, pRb is critical for maintaining the postmitotic quiescent state required for healthy neuronal functions. Inactivation of pRb in neurons leads to loss of function due to a slow but inevitable neurodegeneration process once adult neurons reenter the cell cycle (12, 13). Previous studies from our group have shown that HFD induced pRb phosphorylation in the MBH, including in POMC neurons, and this phosphorylation inactivated pRb, as indicated by expression of E2F target genes in otherwise quiescent neurons (14). Further, conditional deletion of *Rb1* in POMC neurons resulted in cell cycle reentry, neuronal apoptosis, and obesity; however, in contrast, deleting *Rb1* in the antagonizing AgRP/NPY neurons was well tolerated (14). These findings were consistent with several other studies in the field, all of which have demonstrated that POMC neurons are critically required for the maintenance of a normoweight energy balance state, while AgRP/NPY neurons are largely dispensable due to their primary role as POMC neuron antagonists (15–17). We thus proposed a potentially novel mechanism underlying DIO: HFD disrupts MBH neuron homeostasis by phosphoinactivating pRb to compromise the function of MBH neurons, subsequently leading to DIO due to the functional loss of the indispensable POMC neuron population. As such, we hypothesized that inhibiting the phosphoinactivation of pRb during HFD should inhibit DIO. Here, we validated this hypothesis using molecular and pharmacological approaches and, in so doing, obtained a preclinical basis for repurposing FDA-approved cyclin-dependent kinase 4/6 (CDK4/6) inhibitors for obesity management.

## Results

**Figure 1:**
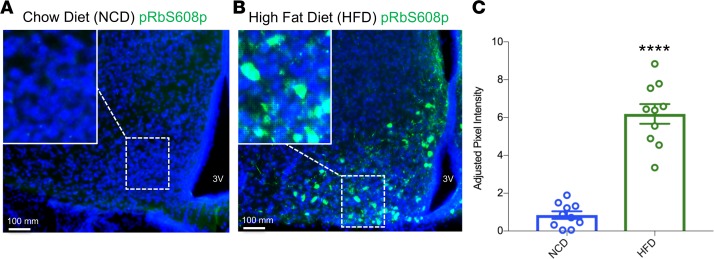
High-fat diet feeding induces pRb phosphorylation in the MBH. Male 6-week-old C57BL/6J mice were fed either (**A**) standard normal chow diet (NCD) or (**B**) high-fat diet (HFD, 60%-Kcal from fat) for 8 weeks. Representative mediobasal hypothalamus (MBH) sections from mice harvested after 8 weeks of HFD or NCD feeding were stained with pRbS608p antibody for phosphorylated pRb. Cell nuclei were counterstained using DAPI. (**C**) Staining intensity quantified using ImageJ, represented as the average of 10 images in each group, 2 images per mouse. 3V, third cerebral ventricle. *n* = 5 per group. Data represent ± SEM. ****P < 0.0001, by 2 tailed Student’s *t* test. Inset magnification, 20×.

**Figure 2:**
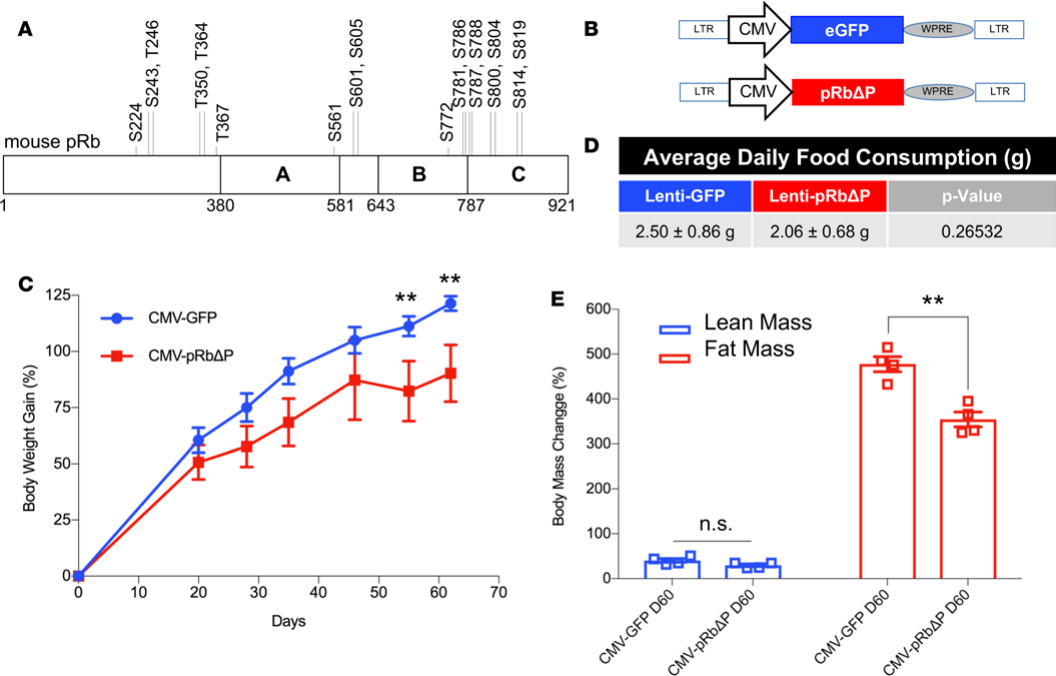
Expressing unphosphorylable pRb in the MBH inhibits DIO. (**A**) Schematic representation of murine pRb protein, with 18 phosphorylation consensus sites (Ser/Thr-Pro) for CDK marked. (**B**) Schematic drawing of lentiviral vectors. Expression cassettes were cloned in pCCL.sin.cPPTs lentiviral backbone. pRbΔP, unphosphorylable pRb, since all 18 Ser/Thr-Pro are mutated to A. WPRE, woodchuck hepatitis virus posttranscriptional regulatory element. (**C**) Two groups of weight and body composition–matched 6-week-old male C57BL/6J were stereotaxically injected in the mediobasal hypothalamus (MBH) with lentivirus-expressing pRbΔP or GFP as marked. High-fat diet (HFD) feeding was initiated on the same day as injection, and weekly body weight measurements were assessed, represented as percent body weight gain over preinjection weight. (**D**) Average daily food consumption, per animal in the GFP or pRbΔP groups as indicated. (**E**) Body composition changes were assessed by MRI for lean and fat masses as indicated after 60 days on HFD after injection (D60) and are shown as percent change of preinjection mass. *n* = 5 per group. Data represent ± SEM. **P < 0.01, by 2-tailed Student’s *t* test.

**Figure 3:**
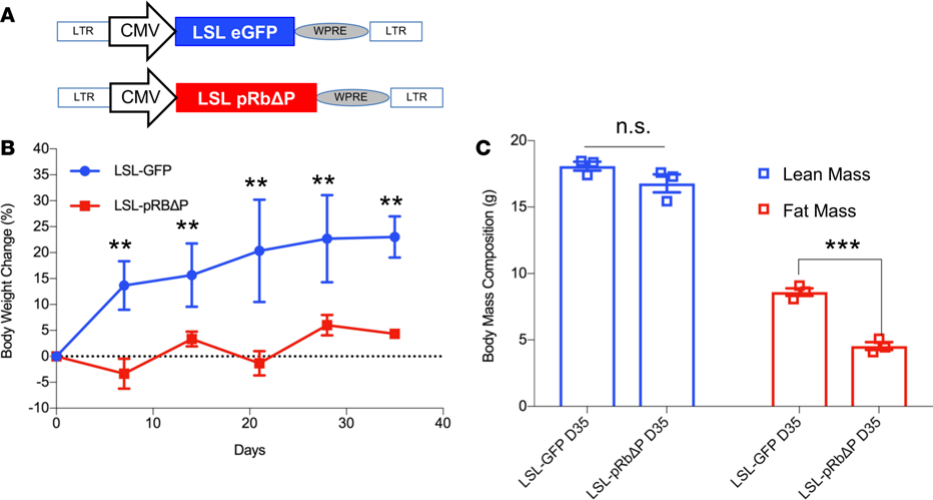
Expressing unphosphorylable pRb conditionally in POMC neurons inhibits DIO. (**A**) Schematic drawing of lentiviral vectors. Expression cassettes were cloned in pCCL.sin.cPPTs lentiviral backbone. pRbΔP, unphosphorylable pRb (all 18 Ser/Thr-Pro are mutated to A). WPRE, woodchuck hepatitis virus posttranscriptional regulatory element. LSL, LoxP-Stop-LoxP for Cre-dependent expression. (**B**) Two groups of weight and body composition–matched 6-week-old female POMC-Cre transgenic mice were stereotaxically injected in the mediobasal hypothalamus (MBH) with lentivirus-expressing LSL-pRbΔP or LSL-GFP as marked. High-fat diet (HFD) feeding was initiated on the same day as injection, and weekly body weight measurements were assessed, represented as percent body weight gain over preinjection weight. (**C**) Body composition changes were assessed by MRI for lean and fat masses as indicated after 35 days on HFD after injection (D35) and are shown as gross grams of fat or lean body mass. *n* = 3 per group. Data represent ± SEM. **P < 0.01, ***P < 0.001, by 2 tailed Student’s t test.

*Expressing unphosphorylablepRb in the MBH or conditionally in POMC neurons inhibits DIO*. Our previous work (14) demonstrates that HFD feeding in C57BL/6J mice resulted in the phosphorylation of pRb in the MBH, leading to subsequent inactivation of pRb and increased E2F target gene expression. This finding was highly reproducible (Figure 1), and we quantified gene expression results by isolating POMC neurons from HFD-fed mice for quantitative PCR (qPCR). We determined that expression of 8 of 10 candidate E2F target genes increased following 8 weeks of HFD feeding (Supplemental Figure 1; supplemental material available online with this article; https://doi.org/10.1172/jci.insight.123000DS1). We next set out to determine the physiological relevance of pRb phosphoinactivation in the MBH to DIO. A murine pRb open reading frame in which 18 CDK-consensus phosphorylation sites (Ser/Thr-Pro, Figure 2A) were changed to alanine (18) was packaged into a lentiviral expression system (Figure 2B) for stereotaxic injection to express unphosphorylable pRb (pRbΔP) in the MBH of 6-week-old C57BL/6J mice on HFD. We confirmed anatomical injection-site success (Supplemental Figure 2) and found that body weight gain on HFD was significantly reduced in mice receiving lenti-CMV-pRbΔP compared with mice receiving lenti-CMV-GFP (Figure 2C). Body mass composition analysis determined that fat mass gain, but not lean mass gain, was significantly reduced in the pRbΔP group at 60 days following lenti-CMV-pRbΔP injection (Figure 2E). Food intake was not significantly reduced (Figure 2D). Although standard DIO mouse models commence HFD feeding at 4–6 weeks of age (19), the C57BL/6J strain concurrently demonstrates a peak animal growth rate between 5 and 9 weeks of age (20). To ensure that the effects of pRbΔP injection on body mass gain were not confounded by fluctuating animal growth rate, a repeat trial utilizing 10-week-old male C57BL/6J mice (with plateaued lean mass growth rate; ref. 20) was performed to confirm the anti-DIO effects of pRbΔP and demonstrated a significant reduction in fat mass 21 days after virus injection (Supplemental Figure 3). Our previous work indicated that pRb is required for the anorexigenic functions of POMC neurons but is dispensable in orexigenic AgRP neurons (14). We therefore determined whether conditional expression of pRbΔP in POMC neurons alone, utilizing a lox-STOP-lox (LSL) expression vector (Figure 3A) and mice expressing tissue-specific Cre-recombinase under the POMC promoter (POMC-Cre) (21), was sufficient for DIO inhibition. We found that LSL-pRbΔP–injected POMC-Cre female mice showed significantly reduced body weight gain (Figure 3B) and fat mass gain (Figure 3C) on HFD 35 days after injection, compared with LSL-GFP control–injected mice (for direct comparison, we noted that POMC-Cre/LSL-GFP–injected mice demonstrated similar degrees of fat mass gain as the C57BL/6J CMV-GFP–injected mice).

### Intracerebroventricular delivery of CDK inhibitors selectively prevents fat mass gain during high-fat feeding.

**Figure 4:**
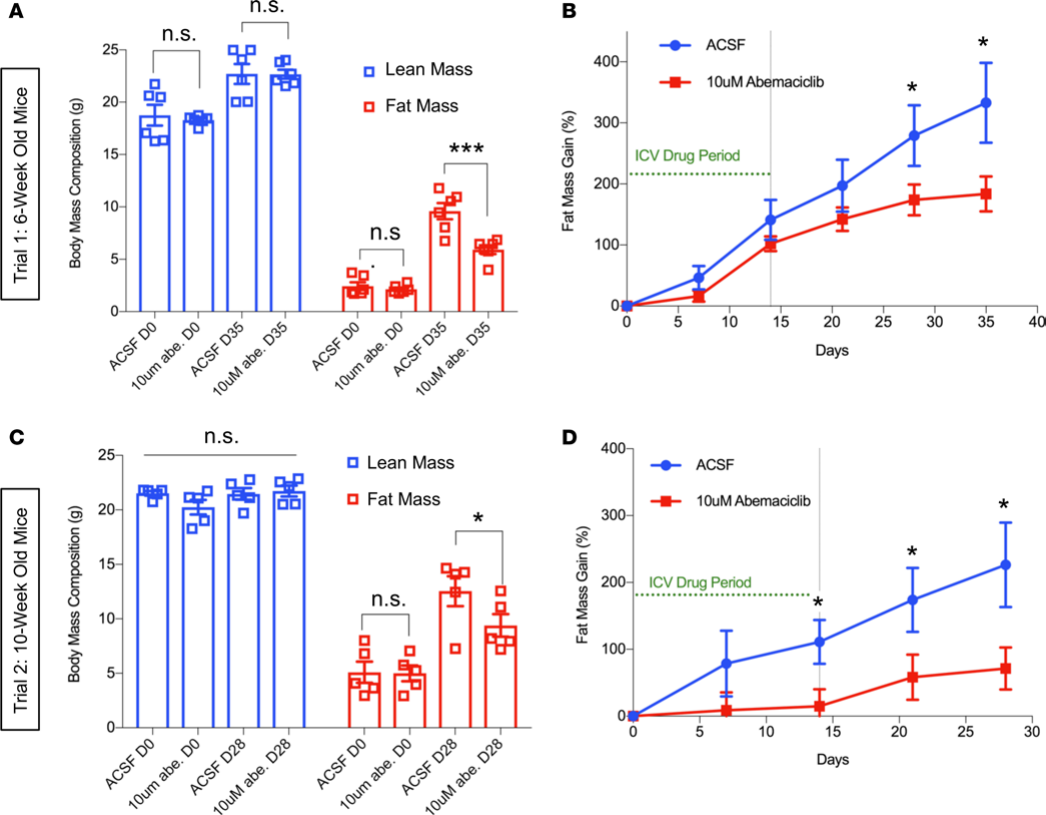
Intracerebroventricular (icv) administration of CDK4/6 inhibitor abemaciclib inhibits DIO. Two groups of weight and body composition–matched male 6-week-old (Trial 1) and 2 groups of weight and body composition–matched male 10-week-old (Trial 2) C57BL/6J mice were stereotaxically cannulated in the third ventricle. High-fat diet (HFD) feeding was initiated on the same day as cannula placement surgery. Daily injections (Monday–Friday) were performed for 2 weeks via icv cannulas to administer 1 μl of 10 μM abemaciclib (abe.) or 1 μl of artificial cerebrospinal fluid (ACSF) control. (**A** and **C**) Body composition at the indicated days after HFD initiation, assessed by MRI. (**B** and **D**) Fat mass gain as percent of presurgery fat masses were measured at 2 weeks after injection start date and 3 (**B**) or 2 (**D**) weeks after conclusion of abemaciclib icv treatment. *n* = 6 per group for **A** and **B,** 5 per group for **C** and **D**. Data represent ± SEM. *P < 0.05, ***P < 0.001 by 2-tailed Student’s *t* test.

As pRb is frequently inactivated by hyperphosphorylation in cancer, using inhibitors of cyclin/CDK complexes to reactivate pRb has been a highly successful rationale for many current cancer therapies (22). Accordingly, we tested the effects of CDK inhibitors delivered by intracerebroventricular injection (icv) on DIO. Dual therapy using CDK4/6 inhibitor abemaciclib and CDK2 inhibitor dinaciclib, delivered daily for 2 weeks, demonstrated a rapid reduction in fat mass gain in animals consuming HFD (Supplemental Figure 4). Clinically, dinaciclib has displayed significant toxicity in a phase I trial (23), while the FDA-approved CDK4/6 selective inhibitor abemaciclib has shown remarkable efficacy in safely treating ER^+^/HER2^–^/RB1^WT^ breast cancer (24, 25). Additionally, monophosphorylation of pRb by cyclin D/CDK4/6 is required for the ensuing hyperphosphorylation and inactivation of pRb by cyclin E/CDK2 (26), which we confirmed in cultured NIH-3T3 cells by comparing CDK4 knockdown by shRNA with abemaciclib treatment (Supplemental Figure 5; see complete unedited blots in the supplemental material). Furthermore, a recent gene expression analysis of the mouse hypothalamus at single-cell resolution demonstrated that CDK4 is abundantly expressed in hypothalamus neurons, but expression of CDK2 and CDK6 is largely undetectable in ARC neuron populations (Supplemental Figure 6; ref. 27). We thus concluded that neuronal CDK4 was the most likely pharmacological target for DIO therapy and initiated trials of abemaciclib icv monotherapy in 6-week-old C57BL/6J mice on HFD. Compared with body composition–matched controls, abemaciclib treatment dramatically lowered fat mass gain and showed no significant effect on lean mass gain, indicating a selective inhibition of fat mass gain with no detrimental effects on animal growth (Figure 4A). Furthermore, mice treated with abemaciclib daily for 2 weeks demonstrated a significantly delayed rate of fat mass gain for up to 3 subsequent weeks of HFD exposure (Figure 4B). As per the pRbΔP study, we performed a second trial utilizing 10-week-old male C57BL/6 mice to control for any effects of abemaciclib on peak animal growth rate between 5 and 9 weeks of age (20). This repeat trial reproduced our finding that abemaciclib icv delivery limited fat mass gain while having no effect on lean mass (Figure 4, C and D). We harvested hypothalamic and adipose tissue from mice in each cohort immediately after completion of abemaciclib treatment and found that treated animals demonstrated a marked reduction of phosphorylated pRb in the MBH (Supplemental Figure 7A). Consistent with reduced fat mass gain in abemaciclib-treated mice, histological examination of gonadal fat pads demonstrated markedly decreased adipocyte hypertrophy in the abemaciclib-treated group (Supplemental Figure 7B).

### CDK inhibitor therapy inhibits DIO by increasing lipid utilization during high-fat feeding.

**Figure 5:**
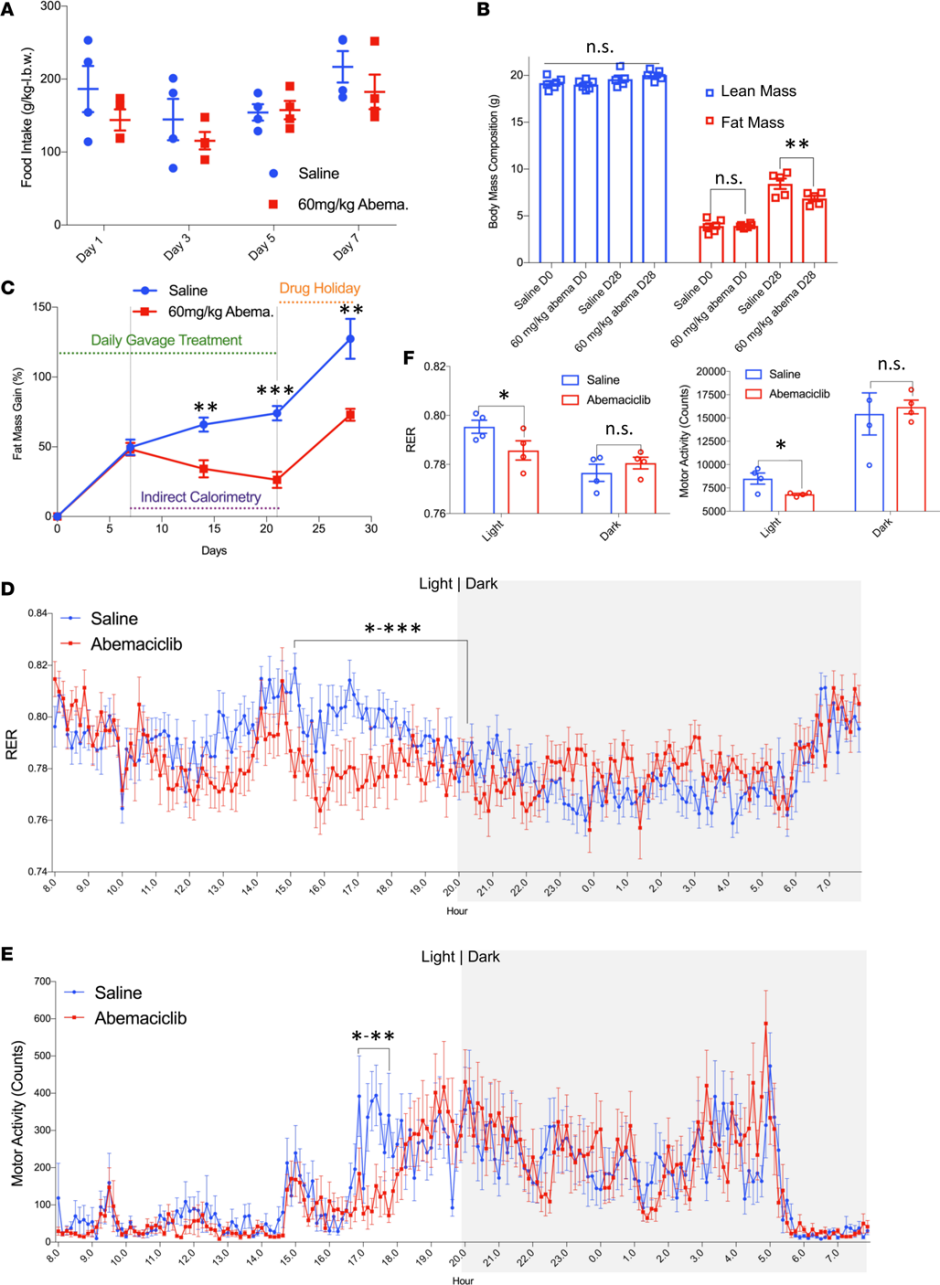
Oral administration of abemaciclib increases lipid utilization efficiency. Two groups of weight and body composition–matched male 6-week-old C57BL/6J mice were gavaged daily with either abemaciclib (60 mg/kg) or saline, with high-fat diet (HFD) feeding initiated on the same day as the first drug treatment. (**A**) Daily food intake was measured at the indicated days during the treatment cycle. (**B**) After 21 days of continuous treatment, drug administration was discontinued for a 7-day “drug holiday,” after which body composition was reassessed on day 28 (D28). Body mass compositions of cohorts were assessed by MRI. (**C**) Fat mass gain as percent of pretreatment fat mass shown during the 21-day drug treatment period and the 7-day “drug holiday.” (**D** and **E**) Twenty-four–hour measurements of respiratory exchange ratio (RER) (**D**) and motor activity (**E**) were assessed by a continuous lab animal monitoring system (CLAMS) during days 7–21 of the drug treatment cycle and are presented as hourly averages over the measurement day period. (**F**) Average values of RER and motor activity during light and dark phase. *n* = 6 per group for (**B**–**C**). However, for calorimetry studies (**A** and **D**–**F**), 4 animals per group were assessed due to technical limitation of 8 available measurement channels. Data represent ± SEM. *P < 0.05, **P < 0.01, ***P < 0.001 by 2-tailed Student’s *t* test. l.b.w. = lean body weight. *P* value ranges between data points over marked areas indicated by *-** and *-***.

With clinical feasibility in mind, we next determined whether oral abemaciclib treatment could recapitulate the DIO inhibition seen in icv abemaciclib treatment. Multiple studies have reproduced the finding that orally administered abemaciclib, at doses comparable with current clinical use, can cross the blood-brain barrier and reach unbound cerebrospinal concentrations several folds above the IC_50_ for CDK4 inhibition (24). In our studies, oral administration of 60 mg/kg abemaciclib did not significantly reduce food intake (Figure 5A), but body composition analysis performed after 21 days of continuous drug treatment followed by 7 days of no drug demonstrated significantly reduced fat mass gain on HFD, with no significant loss of lean mass (Figure 5, B and C). Indirect calorimetry studies assessed continuously during days 7–21 of the treatment cycle uncovered a daily period comprising several hours of significantly reduced respiratory exchange ratio (RER) in the drug-treated cohort (Figure 5D), suggesting that oral abemaciclib treatment promotes more efficient utilization of lipid as an energy source and explaining, in part, the reduced fat mass gain in this group. Importantly, this period of reduced RER coincided with a period of reduced motor activity immediately prior to the onset of the dark phase (Figure 5E), when rodents become active to engage in food-seeking behavior. Importantly, comprehensive analysis of the indirect calorimetry data (Figure 5F) was able to demonstrate an increased fat utilization efficiency of 3.24% ± 1.58% (Table 1) in the abemaciclib-treated group, suggesting that abemaciclib treatment limited fat mass gain through increased lipid oxidation. Daily and cumulative food intake differences were not statistically significant between the groups (Table 1). Thus, it is likely that abemaciclib treatment inhibited DIO largely through the cumulative effect of a short daily period of increased lipid utilization efficiency. Importantly, we saw no effects on body mass composition when animals on a chow diet were continuously treated with oral abemaciclib (Supplemental Figure 8) for up to 42 days, suggesting that CDK4 inhibition corrects HFD-mediated neuronal injury without detrimental effects on the basal energy balance circuit during normal, non-HFD.

### CDK inhibitor therapy selectively reduces fat mass to treat DIO.

**Figure 6:**
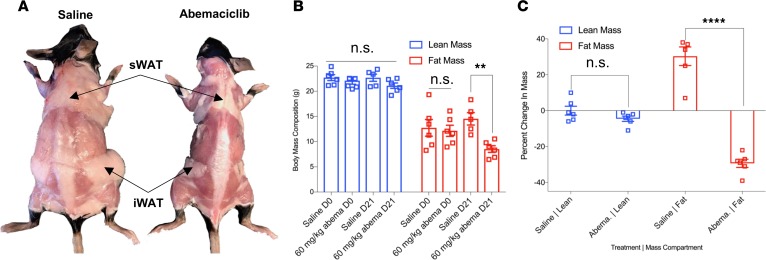
Oral administration of abemaciclib selectively reduces fat mass in DIO mice. Two groups of weight and body composition–matched male 9-week-old C57BL/6J mice were fed high-fat diet (HFD) for 4 weeks and rematched into pairs based on similar post-HFD body mass compositions. HFD feeding was continued, and animals were administered continuous daily treatment with 60 mg/kg abemaciclib or saline control by gavage. (**A**) Saline- and drug-treated animals at 21 days after treatment. Inguinal white adipose tissue (iWAT) and posterior s.c. WAT (sWAT) depots indicated by arrowheads. (**B**) Body mass compositions of cohorts at after 21 days of continuous treatment, assessed by MRI. (**C**) Fat and lean mass changes as percent of pretreatment mass after 21 days of continuous treatment. *n* = 6 per group. Data represent ± SEM. **P < 0.01, ****P < 0.0001 by 2-tailed Student’s *t* test.

**Figure 7:**
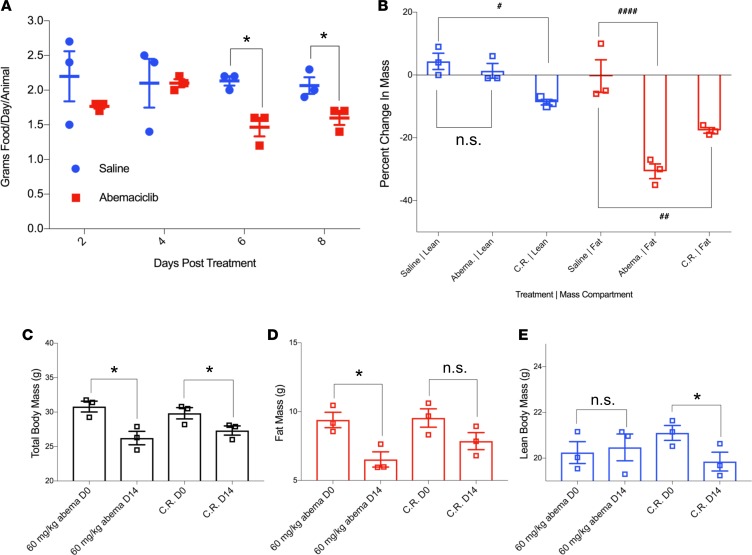
Abemaciclib treatment leads to selective loss of fat mass in DIO mice. Two groups of weight and body composition–matched male 9-week-old C57BL/6J mice were fed HFD for 4 weeks and rematched into pairs based on similar post-HFD body mass compositions. HFD feeding was continued, and animals were administered continuous daily treatment with 60 mg/kg abemaciclib or saline control by gavage. (**A**) Daily food intake was measured at the indicated days after treatment initiation. (**B**) Fat and lean mass changes as percent of pretreatment masses was assessed by MRI after 14 days of treatment. C.R. indicates calorically restricted, in which a group of mice had their food intake titrated down to reduce total body mass to match the abemaciclib-treated group. (**C**) Total body, (**D**) fat mass, and (**E**) lean mass of abemaciclib-treated versus food-restricted C.R. animals were assessed by MRI after 14 days of treatment or food restriction (C.R. group only). Note equivalent reductions in total body weight but significant fat mass reduction only in abemaciclib group and significant lean mass reduction only in food restricted group. *n* = 3 per group. Data represent ± SEM. *P < 0.05, by 2-tailed Student’s *t* test. ^#^*P* < 0.05, ^##^*P* < 0.01, ^####^*P* < 0.0001, by parametric 1-way ANOVA with Bonferroni correction.

**Figure 8:**
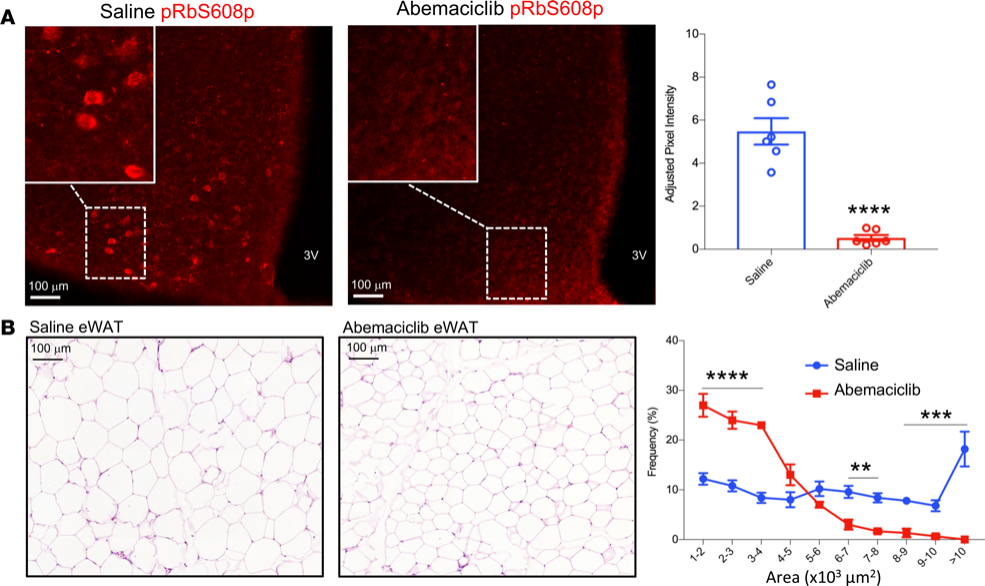
Oral administration of abemaciclib reversed pRb phosphorylation in the MBH and adipocyte hypertrophy in DIO mice. Two groups of weight and body composition–matched male 9-week-old C57BL/6J mice were fed HFD for 4 weeks and rematched into pairs based on similar post-HFD body mass compositions. HFD feeding was continued, and animals were administered continuous daily treatment with 60 mg/kg abemaciclib or saline control by gavage. (**A**) Representative MBH sections from mice harvested upon conclusion of 21 days of abemaciclib or saline gavage treatment were stained with pRbS608p antibody for phosphorylated pRb. Staining intensity is quantified at right. 3V, third cerebral ventricle. (**B**) H&E-stained gonadal fat pads from same mice. Frequency distribution of adipocyte sizes quantified at right. *n* = 6 per group. Data represent ± SEM. **P < 0.01, ***P < 0.001, ****P < 0.0001 by 2-tailed Student’s *t* test. Inset magnification, 20×.

Clinically, pharmacologic appetite suppression, caloric restriction, or bariatric surgery can acutely reduce body weight in obese patients via negative energy balance (28). We set out to determine whether oral abemaciclib could selectively reduce fat mass in mice that already developed DIO. We found oral abemaciclib treatment rapidly reduced fat mass in obese mice, while having no effect on lean mass (Figure 6). Although intermittent differences in food intake after abemaciclib treatment were observed, these were transient events that could not be continuously observed on a daily basis (Figure 7A). Moreover, cumulative weekly food intake differences were not found to be statistically significant between abemaciclib-treated and saline control groups (Table 2). In further investigating the role of food intake in abemaciclib-mediated loss of fat mass, we determined that 14 days of daily abemaciclib treatment was in fact more efficacious in reducing body fat mass than restricted food intake for 14 days. Oral abemaciclib treatment reduced total body mass and fat mass but had no significant effect on lean mass (Figure 7B). In comparison, restricted feeding to weight-match abemaciclib-treated animals (50% reduction of the mean ad libitum food intake of saline-treated controls in Figure 7A) significantly reduced both fat mass and lean mass compared with saline-treated groups. However, the reduction in gross grams of fat mass in the food-restricted group was not statistically significant when compared with the fat loss of the abemaciclib-treated group (Figure 7, C–E). Therefore, we confirmed that abemaciclib treatment results in the selective loss of fat mass without the obligatory loss of lean mass, which occurs during restricted food intake, and this — combined with cumulative food intake data (Table 2) — suggested that abemaciclib treatment lowered fat mass in obese animals largely through increased fat utilization, as opposed to decreased fat consumption. We next confirmed whether oral abemaciclib treatment inhibited pRb phosphorylation in the MBH when animals were kept on HFD. We found abemaciclib-treated animals demonstrated markedly reduced phosphorylation of pRb in the MBH in postmortem analysis (Figure 8A), as well as a significant reduction in adipocyte hypertrophy (Figure 8B) and hepatosteatosis (Supplemental Figure 9). Importantly, this loss of fat mass was observed in every individual animal in the treated cohort, regardless of pretreatment fat mass (Supplemental Figure 10).

## Discussion

Current medical and surgical obesity management treatments all involve varying methods of calorie restriction leading to negative energy balance and are, thus, accompanied by the undesirable but obligatory loss of lean skeletal muscle mass (29). This results in serious patient side effects such as fatigue, malaise, and other hindrances on patient quality of life. Furthermore, neurostimulant appetite-suppressant drugs such as phentermine/topiramate (30) or sibutramine (31) produce only modest weight loss in patients and are unfeasible for long-term sustained use, precluding their utilization for forms of morbid obesity that requires years of continuous therapy. In our study, the CDK4/6 inhibitor abemaciclib distinguishes itself with the capacity to correct fat mass imbalances in DIO with no effect on lean mass, forecasting a highly attractive and potentially novel pharmacological intervention for obesity management. While three CDK4/6 inhibitors are in fact currently FDA approved for life-long therapeutic use to treat metastatic breast cancer, their time on the market has been limited to only the last 3 years. As such, long-term effects in patients have yet to be fully determined (32).

Moreover, all patients currently undergoing CDK4/6 inhibitor therapy are diagnosed with metastatic breast cancer, a chronic condition that negatively affects energy balance factors, such as appetite, fatigue, and malaise (33), and necessitates chemotherapy regimens of drugs with negative side effects on energy balance, such as nausea, early satiety, and delayed gastric emptying (34). As such, a large-scale clinical trial in obese patients without cancer is critical to determine the safety and efficacy of CDK4/6 inhibitors solely as an antiobesity therapeutic.

Another limitation of this study is that the exact molecular pathway by which circulating HFD metabolites activate the CDK4 pathway to phosphorylate pRb in the hypothalamus requires further study. Although cyclin/CDK complexes are required to complete the hyperphosphorylation necessary to inactivate pRb, exactly how circulating HFD metabolites induce their activity remains to be determined. The possible downregulation of endogenous cyclin/CDK inhibitors such as p16, p21, and p27 directly by HFD intake may potentiate pRb inactivation in the hypothalamus and warrants further study.

While pRb phosphoinactivation due to HFD occurs globally in the MBH (14), how this phenomenon selectively compromises the function of the anorexigenic POMC neuron population remains unclear. It has been shown that neither AgRP nor NPY is critically required for energy homeostasis, as mice with deletion of either gene or both genes are phenotypically and metabolically indistinguishable from WT mice (17). Meanwhile, the genetic deletion of POMC alone is sufficient to produce massive obesity in mice (15), which is phenocopied in mice harboring the dual deletion of AgRP and POMC (16). These studies suggest that, while POMC neurons are vitally required in preventing obesity, the primary role of AgRP/NPY neurons is to serve as POMC antagonists. However, if AgRP neurons differ from POMC neurons in that their function is not compromised by pRb phosphoinactivation, unopposed AgRP/NPY signaling during HFD may drive increased food-seeking behavior to cause DIO (35, 36). In this context, DIO is prevented via the restoration of POMC neuron function by CDK4 inhibitors reducing pRb phosphoinactivation in POMC neurons. In summary, several further studies utilizing CDK4 inhibitor treatment in mice genetically lacking POMC, its receptor MC4R, or AgRP/NPY (alone and in and combination) can shed further light on the differential roles of these neuropeptides in potentiating CDK4 inhibitor therapy to prevent and treat DIO.

Interestingly, POMC reactivation in obese Pomc-null mice can normalize leptin sensitivity and energy balance after weight loss by caloric restriction (37). These findings suggest that restoration of POMC signaling can reverse obesity. Furthermore, inducing expression of POMC in the hypothalamus has been directly linked to upregulated peripheral lipolysis in white adipose tissue (38). In MBH neurons, the POMC peptide is processed into the biologically active MSH, an activating ligand for the central melanocortin receptor MC4R. MC4R, however, is also found on preganglionic neurons of the sympathetic nervous system (39), linking POMC neuron activation to increased rates of lipolysis due to increased adrenergic signaling to white adipose tissue (40). While these direct links between POMC activity and peripheral lipolysis provide an attractive mechanism of action for the fat-mass–selective effects of CDK4/6 inhibitors demonstrated in our studies, the complex underlying neurophysiological axis between HFD intake, neuronal homeostasis, and peripheral fat metabolism requires further analysis.

Although our findings support a central mechanism of action for the antiobesity effects of CDK4/6 inhibitors, studies in the field have indicated additional roles for CDK/cyclin complexes in the peripheral regulation of adipose tissue. It has been shown that CDK6 can inhibit the transition of white to beige adipose tissue through phosphorylation-mediated degradation of the transcription factor RUNX1 (41), indicating CDK4/6 inhibitors may decrease adipose tissue mass by converting white adipose tissue to thermogenic brown fat, in a wholly pRb independent manner. However, these findings directly conflict with other studies in the field, which have shown that pRb directly suppresses the white to brown adipose transition and thermogenesis process, suggesting that the activation of pRb in adipose tissue via CDK4/6 inhibitors would more likely prevent fat mass loss (42). In short, the role of non-pRb targets of CDKs such as RUNX1 and pRb itself in adipose tissue regulation still remains largely unexplored and requires further study to determine how CDK4/6 inhibitor therapy may be affecting peripheral adipose tissue during DIO.

Our study demonstrates that targeted inhibition of central CDK4 can decrease fat mass while preserving lean mass in obese mice during HFD feeding. This effect occurs without reducing food intake but by accelerating lipid oxidation within a restricted time period during the circadian cycle, leading to preferential loss of fat mass. Importantly, CDK4/6 inhibitors do not affect body composition or body weight of mice on normal chow diet, indicating that their mechanism of action during DIO treatment is the restoration of POMC neuron function as a critical constituent of the body’s weight change threshold detection mechanism (43). As a corollary of our model for the mechanism of CDK4/6 inhibitors in treating DIO, normal-weight animals on normal chow diet do not show any effects from long-term CDK4/6 inhibitor treatment, most likely due to the fact that pRb is active and in its hypophosphorylated form in these animals. Thus, long-term high-fat feeding that leads to the hyperphosphorylation and inactivation of pRb in POMC neurons could contribute to the resistance of overweight and obese individuals to long-term maintenance of a reduced body mass; this potentially novel model of DIO necessitates further study, as well. Currently, three CDK4/6 inhibitors are already FDA approved for clinical use. Our study provides the preclinical basis for the expedient repurposing of CDK4/6 inhibitors into clinical trials for obesity management.

## Methods

### Materials.

Materials, reagents, and resources are listed in Supplemental Table 1. Note that CDK4 inhibitor and CDK4/6 inhibitor are used interchangeably in this study. All current FDA-approved CDK inhibitors of CDK4 also target CDK6, but CDK6 is largely unexpressed in hypothalamus neurons (Supplemental Figure 6) and is, thus, precluded from being a molecular target in the context of this study.

### Study design.

Our study objective was to determine the functional relevance of HFD-induced phosphoinactivation of pRb in the MBH to DIO. We used parallel genetic and pharmacological approaches to determine if pRb phosphoinactivation was casual to DIO and if subsequent reactivation could ameliorate DIO. We injected lentivirus into the MBH of mice on HFD to express pRbΔP and found that this was sufficient to prevent DIO. As pRb is intracellularly inactivated by CDKs, the reactivation of pRb can be achieved utilizing small molecule inhibitors of CDK/cyclin complexes. We determined CDK4 to be the major kinase for pRb phosphorylation in the brain and determined the effect of CDK4/6 inhibitor abemaciclib delivered by icv in preventing DIO. In the context of clinical feasibility, we next tested the effect of daily oral abemaciclib treatment, at doses comparable with FDA-approved human use, for treating DIO. We used indirect calorimetry studies to determine the effect of CDK4/6 therapy on fat oxidation, food-seeking behavior, and how this was able to prevent DIO. Clinical feasibility was further expanded by testing the effect of abemaciclib therapy on mice that already developed DIO and determined that oral abemaciclib therapy lowered fat mass in obese animals without affecting lean mass. Investigators who performed mouse experiments and tissue histology were blinded to experimental groups.

### DIO animal models.

This study largely utilized male C57BL/6J mice, which were purchased directly from the Jackson Laboratory. It has been shown that, although C57BL/6J-strain mice are widely used DIO models, significant numbers of animals in this strain may be naturally resistant to DIO (DIO-R) (44). To identify DIO-R mice, 6- to 9-week-old C57BL/6J mice were given HFD for 4 weeks. Mice exhibiting body weight or fat mass (measured as %fat of whole body composition) 1 SD below the Jackson Laboratory standards for DIO parameters at 8 weeks age (28.1 ± 2.1 g body weight, 30.0% ± 1.8% fat) were excluded from our studies. Additional heterogeneity in varying degrees of obesity among individual animals was addressed by the experimental and control cohorts containing animals individually match-paired by pretreatment body mass and body composition (i.e., a near-identical distribution, mean, and variance in pretreatment body weights and mass-compositions in obese control and experimental groups). As this prescreen was not technically feasible when studies were commenced on 6-week-old mice (as it would be difficult to parse if mice younger than 6 weeks were gaining mass due to HFD or normal growth), an alternate screening method was utilized: the only statistically significant predictor of a DIO-R phenotype was low body weight at 6 weeks of age (prior to HFD initiation) (44). All cohorts used as described consisted of age-matched animals ordered from Jackson Laboratory, of which only the animals in the upper 75th–80th percentile of body weight were used for subsequent study (i.e., 15 mice ordered for a trial, 3 of lowest body weight discarded, study *n =* 12. As before, each experimental arm contained animals individually match-paired by pretreatment body mass and body composition. Mice used in the presented experiments were WT C57BL/6J male from Jackson Laboratory (stock no. 000664). POMC-Cre mixed strain mice were obtained from Jackson Laboratory (stock no. 010714) and back-crossed with C57BL/6J-strain mice 4 times prior to experimental use. POMC-Cre mice were crossed with a Rosa-EYFP reporter strain from Jackson Laboratory (stock no. 006148) for POMC neuron isolation experiments (Supplemental Figure 1).

### Lentivirus production.

Various lentiviral plasmids were used by the Einstein Gene Therapy Core to generate high-titer virus stocks. 293T cells were transfected using standard calcium phosphate protocol with vector plasmid and various helper/packaging plasmids. Virus was collected and concentrated by ultracentrifugation, and titer was determined by using LentiX PCR based tittering kit (Clontech, PT4006-1). Knockdown vectors were designed as described in ref. 45.

### Stereotaxic intra-MBH injection and ventricular cannulation.

Mice were maintained under isoflurane anesthesia and placed in a stereotaxic apparatus. For intra-MBH injection, 0.3 μl of viral stocks containing 5,000–15,000 transducing units were bilaterally injected into the ARC (from bregma: anterior-posterior [AP], –1.3 mm; medial-lateral [ML], ± 0.1 mm; dorsal-ventral [DV], –6.0 mm). The operators were blinded to the group allocation during the experiment. For ventricular cannulation, sterile custom guide cannulas were stereotaxically implanted into the third ventricle under aseptic conditions (from bregma: AP, –1.2 mm; ML, 0 mm; DV, –5.5 mm) for local injection of drugs.

### FACS.

POMC-Cre (46) or RosaR(eYFP) mice (21) have been described. Genotyping primers for POMC-Cre;RosaR(eYFP) mice are listed in Supplemental Table 1.

Freshly dissected hypothalamus tissue was dissociated into single cell suspensions using the Papain Dissociation System (Worthington, LK003150) at manufacturers recommendations. For tissue extracted from POMC-Cre;RosaR(eYFP) mice, cell suspensions were FACS sorted on a DakoCytomation MoFlo (Einstein Flow Cytometry Core), using gating parameters as shown in Supplemental Figure 1, A–C.

### qPCR.

RNA was isolated from FACS sorted cells or homogenized hypothalamic tissue, treated with DNAse I (New England Biolabs) for 30 minutes and purified using the RNeasy Mini Kit (Qiagen). All reverse transcriptase reactions were carried out with the Verso cDNA Synthesis Kit (Thermo Fisher Scientific), according to the manufacturer’s protocol. Primers (as listed above) for different genes with annealing temperature of 60°C for all were chosen with the Primer3 program. The expression level of GAPDH was used as an internal control. For qPCR, 20 ng cDNA were mixed with 1× Choice Taq Blue Mastermix (Denville Scientific Inc.) and 0.5 mM primers and amplified 23–33 cycles. Real-time PCR was performed in triplicate for each gene. Each SYBR Green assay was performed in a 12 μl total reaction volume that included 6 μl of 2× SYBR Green Power master mix (Applied Biosystems), 250 nM of each primer, and 20 ng of template cDNA. Assays were run on a 7500FAST instrument (ABI) under standard conditions recommended by the manufacturer and were 95°C for 10 minutes, followed by 40 cycles of 95°C for 15 seconds and 60°C for 1 minute, followed by melting curve analysis. Data were analyzed using 7500 ABI software, V2.0.6. Fold difference in gene expression was determined by the ΔΔCt method.

### Oral administration of abemaciclib.

Mice were administered drug by gavage without anesthesia using 20 g × 2.25 mm curved animal feeding needles. Appropriate volume of abemaciclib (100 mg/ml stock, in sterile water) was dissolved in a total volume of 300 μl of 0.9% saline supplemented with 1% hydroxyethyl cellulose to achieve a 60 mg/kg body weight/dose.

### Body composition analysis.

Body composition analysis was performed using an EchoMRI-100 Body composition analyzer. Mice were not sedated during analysis.

### Immunofluorescence.

Mice were anesthetized with isoflurane and transcardially perfused sequentially with PBS and 10% neutral buffered formalin. Brains were dissected out and incubated in 4% paraformaldehyde overnight, followed by 48 hours in 30% sucrose solution. Brains were then flash-frozen, and sections were sliced on a Microm HM450 sliding microtome at 50 μm. For immunofluorescence staining, sections were washed in PBS and blocked in 0.1 M PBS buffer containing 0.1% triton X-100, 5% normal goat serum or normal donkey serum, and 5% BSA (MilliporeSigma) for 2 hours at room temperature and then incubated with primary antibodies (diluted 1:200 in blocking buffer) for 72 hours at 4 degrees (Supplemental Table 1). Sections were washed 3 times in PBS and incubated with secondary antibodies (1:500) for 2 hours at room temperature. Tissues were washed, dried, and mounted with VECTASHIELD media containing DAPI. Fluorescence images were obtained with a Nikon Eclipse TE 2000-S fluorescence microscope. Image fluorescence was quantified using ImageJ (NIH).

### Histological analysis.

Gonadal fat pads or whole liver lobes were extracted from sacrificed mice and fixed in 10% neutral buffered formalin. Fixed tissue was sent to the Einstein Histopathology Core for routine staining and sectioning (5-μm sections of paraffin embedded tissue, stained with H&E). For adipose tissue, stained slides were scanned using a 3DHistec Panoramic 250 Flash II slide scanner. Up to 5 random sections per slide were sampled and quantified using ImageJ and Volocity 6.3.

### Cell culture.

NIH-3T3 cells were cultured in DMEM supplemented with 10% FBS.

### Western blotting.

NIH-3T3 cells were homogenized in RIPA buffer to obtain total protein extract, which was separated on SDS-PAGE, blotted, and probed with antibodies listed in Supplemental Table 1, as per manufacturer’s recommendations. Appropriate HRP-conjugated secondary antibodies were used to detect protein expression via chemiluminescence.

### Indirect calorimetry studies.

Columbus Labs comprehensive lab animal monitoring systems (CLAMS) was used to collect multiparameter metabolic data, including food intake, meal sizes, and intervals (which reflect satiety and feeding drive, respectively), physical activity as horizontal and vertical beam breaks, O_2_ consumption, and CO_2_ production to calculate RER. Data was continuously collected for 7 days.

### Statistics.

GraphPad (Prism 7.0c, GraphPad Software) was used to perform statistical tests and plot results. Data are presented as mean ± SEM. *P* values for all data were determined by 2-tailed, unpaired student’s *t* test, and a *P* value less than 0.05 was considered to be significant. For comparison of multiple groups, 1-way ANOVA analysis was performed, with Bonferroni correction applied. For most experiments, between 3 and 6 mice were used per experimental group. Sample sizes for experiments were determined to be appropriate by the current standard used for rodent studies in integrative physiology and metabolism experiments, based on the minimal number of mice required to detect significance using a 2-tailed α of 5% and a power of 0.80. Animals that died during the course of experiments had all predemise data excluded from analysis, and experimental *n* was adjusted accordingly during statistical analysis. Animal studies were replicated at least 2 times. Findings were demonstrated to be reproducible. Data from several repeat experiments included in Supplemental Figures.

### Study approval.

All procedures were reviewed and approved by Einstein Animal Care Committee, conforming to accepted standards of humane animal care, under institutional protocol number 20151213.

## Author contributions

NJI contributed Figures 1–8 and Supplemental Figures 4–10. SML contributed to the construction of lenti-CMV-pRbΔP. ZL contributed Figure 1, C and D, and Supplemental Figures 1–3. GJS directs the Einstein Diabetes Center Animal Physiology Core that performed intra-MBH stereotaxic injections, icv injections, and indirect calorimetry studies. SC and LZ conceived, designed, and supervised the studies. NJI, LZ, and SC wrote the manuscript, and all authors critically reviewed and edited the manuscript.

## Supplementary Material

Supplemental data

## Figures and Tables

**Table 2:**
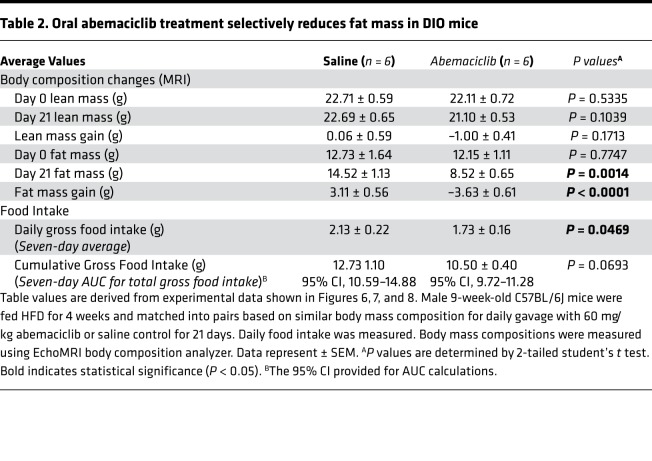
Oral abemaciclib treatment selectively reduces fat mass in DIO mice

**Table 1:**
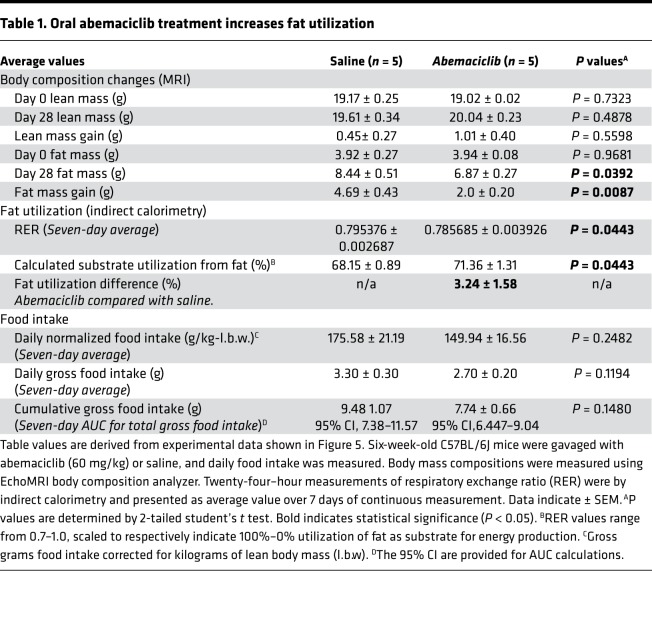
Oral abemaciclib treatment increases fat utilization

## Version 1 09/06/2018

Electronic publication
