# Supplementary material for: Cyclin-dependent kinase 4 is a preclinical target for diet-induced obesity
Source: JCI Insight. 2018 Sep 6;3(17):e123000. doi: 10.1172/jci.insight.123000 (PMC6171799; doi:10.1172/jci.insight.123000)
Supplement: Supplemental data [file jciinsight-3-123000-s129.pdf]

Supplemental Materials for:

Cyclin-dependent kinase 4 is a preclinical target for diet-induced obesity

Authors: Niloy Jafar Iqbal[1], Zhonglei Lu[1,†], Shun Mei Liu[2], Gary J Schwartz[2], Streamson Chua Jr[2*], and Liang Zhu[1.2*]

Affiliations:
[1]Department of Developmental and Molecular Biology, Albert Einstein College of Medicine, Bronx, NY, USA.
[2]Department of Medicine, Albert Einstein College of Medicine, Bronx, NY, USA.

[*]Correspondence to: Streamson Chua (streamson.chua@einstein.yu.edu) or Liang Zhu (liang.zhu@einstein.yu.edu), Albert Einstein College of Medicine, 1300 Morris Park Ave, Bronx NY 10461.

[†]Current address: College of Biological Science and Engineering, Fuzhou University, Fuzhou, Fujian 350108, China.

This PDF file contains:

Supplementary Table 1. Reagents and Resources

Supplementary Figures 1 to 10

Table S1. Reagents and Resources

Reagent or Resource	Vendor	Identifier
**Chemicals**		
Abemaciclib (**LY2835219)**	MedChem Express	Cat# HY-16297
Dinaciclib (**SCH 727965**)	MedChem Express	Cat# HY-10492
Artificial CSF (ACSF)	Tocris	Cat# 3523
High Fat Diet (60%kCal-Fat)	OpenSource Diets	Cat#D12492
**Antibodies**		
Anti-Phospho-pRb S807/S811	Cell Signaling Technologies	Cat#8516
Anti-Phospho-pRb S780	Cell Signaling Technologies	Cat#8180
Anti-Phospho-pRb S608	Cell Signaling Technologies	Cat#8147
Retinoblastoma Protein (total)	Cell Signaling Technologies	Cat#9309
Goat Anti-Rabbit Alexa488	ThermoFisher	Cat#A-11034
Goat Anti-Rabbit Alexa594	ThermoFisher	Cat#A-11037
Anti-CDK4	Santa Cruz Biotechnology	Cat#SC-23896
Anti beta-Actin	Sigma Aldrich	Cat#A5441
Donkey Anti Rabbit-HRP	GE Healthcare	Cat#NA934V
Sheep Anti Mouse-HRP	GE Healthcare	Cat#NA931V
**Viral Vectors and Preparations**		
pCCL.sin.PPTs.CMV.eGFP.WPRE	Zhu/Chua Labs	n/a
pCCL.sin.PPTs.CMV.pRbΔP.WPRE	Zhu/Chua Labs	n/a
pCCL.sin.PPTs.CMV.shEmpty-mCherry.WPRE	Zhu/Chua Labs	n/a
pCCL.sin.PPTs.CMV.shCDK4-mCherry.WPRE	Zhu/Chua Labs	Target Sequence:<br>CCTAGCTAGAATCTACAGCTA
**qPCR Primers**		
P107<br>Fwd: GCAGTCACCACGCCTGTAGC<br>Rev: TTCCCATAGGATTCCGCATAC	***P130***<br>Fwd: CCACTGACTGGCGTGAGGTA<br>Rev: CATCTGGCTGGAAATGCTGA	***E2f1***<br>Fwd: TCACTAAATCTGACCACCAAACG<br>Rev: TTGGACTTCTTGGCAATGAGC
CycE<br>Fwd: GACGTTCTACTTGGCACAGGA<br>Rev: ACACAATGGTCAGAGGGCTTA	***Pcna***<br>Fwd: TGAAGAAGGTGCTGGAGGCT<br>Rev: TTGGACATGCTGGTGAGGTT	***Mcm3***<br>Fwd: GCTTTGCCATTGGGTAGTTC<br>Rev: CGCAGGCGTGAGTATTCTTC
Apaf-1<br>Fwd: GAGCACTCGGAGCAAGTCAAT<br>Rev: AAAGCCTTAAAGTCCCGTCAG	***Casp3***<br>Fwd: GTCTGACTGGAAAGCCGAAAC<br>Rev: GACTGGATGAACCACGACCC	***Casp7***<br>Fwd: GGCGTGACACCCATAAAGGA<br>Rev: CCTGGAACCGTGGAGTAAGC
Puma<br>Fwd: GGTCCAGACTGTGAATCCTGTG<br>Rev: TCCTCCCTCTTCTGAGACTTCC	***Trp73***<br>Fwd: AACGCCGAGCATCAATCC<br>Rev: AGCCCAGACTCTGAGCACTT	***Bim***<br>Fwd: ATCTTGTTGGGCTTACTTGTG<br>Rev: GTCCTGCCTGGTCTTGAAAT
Bad<br>Fwd: CCGAAGGATGAGCGATGAGT<br>Rev: CGGGATGTGGAGCAGAAGAT	***Casp9***<br>Fwd: CCGTGGACATTGGTTCTGG<br>Rev: TCTTGGCAGTCAGGTCGTT	***Bax***<br>Fwd: AGGATGCGTCCACCAAGAA<br>Rev: CAAAGTAGAAGAGGGCAACCAC
POMC<br>Fwd: CAGTGCCAGGACCTCACC<br>Rev: CAGCGAGAGGTCGAGTTT	***AgRP***<br>Fwd: CGGAGGTGCTAGATCCACAGA<br>Rev: AGGACTCGTGCAGCC TTA CAC	***Gapdh***<br>Fwd: GGTTGTCTCCTGCGACTTCA<br>Rev: GGTGGTCCAGGGTTTCTTAC
**Genotyping Primers**		
**Pomc-Cre** mice (Balthasar et al, 2004) were genotyped with the following primers:<br>5'-TGGCTCAATGTCCTTCCTGG-3' and<br>5'-GAAATCAGTGCGTTCGAACGCTAG A-3'<br>The product size is about 580 bp.	**RosaR(eYFP)** mice (Srinivas et al, 2001) were genotyped with the following primers:<br>5'-AAGTTCATCTGCACCACCG-3' and<br>5'-TGCTCAGGTAGTGGTTGTCG –3'.<br>The product size is about 400 bp**.**	

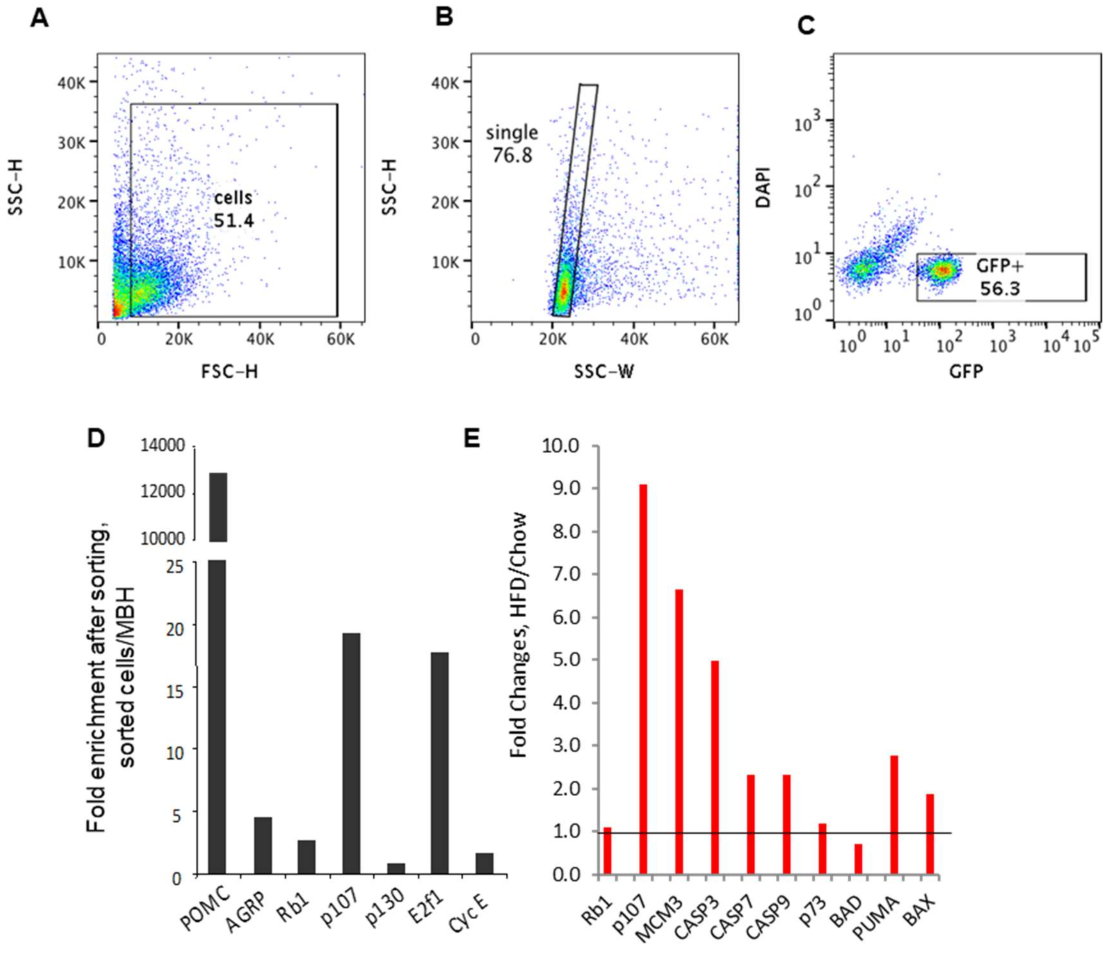

Supplemental Figure 1. E2F target gene expression is higher in POMC neurons following 8 weeks on HFD. GFP based FACS sort was used to purify POMC neurons from dissected MBH in Pomc-Cre;RosaR(eYFP) mouse. (**A**, **B** and **C**) diagrams showing the gates placed successively leading to collection of GFP+ cells. (**D**) RT-qPCR of the purified POMC neurons and an intact MBH, showing fold enrichment comparing sorted cells and intact MBH for the indicated genes. (**E**) RT-qPCR to measure expression of candidate E2F target genes as Fold Changes comparing 8 weeks of HFD over Chow. Expressions were normalized by GAPDH.

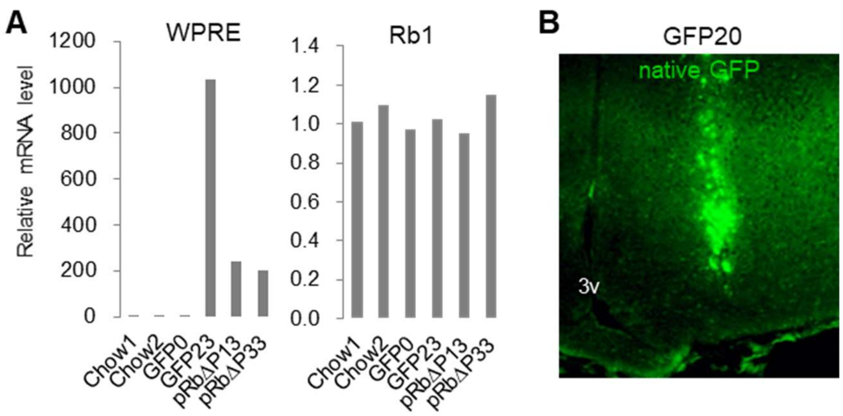

Supplemental Figure 2. Validation of stereotaxic intra-MBH injections success. (**A**) RT-qPCR measuring gene expression from injected lentiviruses in MBH. Presence of WPRE sequence in mRNA indicates viral origin. PCR primers for Rb1 recognize endogenous Rb1 and exogenous RbΔP mRNA. (**B**) Section of another GFP injected MBH (mouse GFP20), showing native GFP fluorescence. 3v, third ventricle.

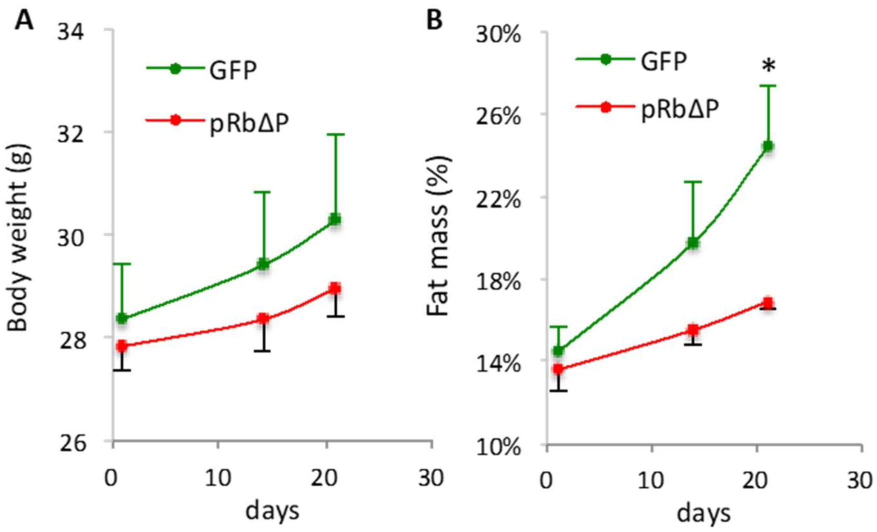

Supplemental Figure 3. Expressing un-phosphorylable pRb in MBH inhibited DIO. Effect of intra-MBH expression of pRbΔP or GFP as marked in WT 10-week old male C57BL/6J mice on body weight gain over pre-HFD weights. Body weight (**A**) and fat mass % (**B**) measurements were taken on days 1, 14, and 21. N = 5 for both GFP and pRbΔP groups. Mean and SEM are shown. ***,** $p < 0.05$ by two tailed Student's *t* test.

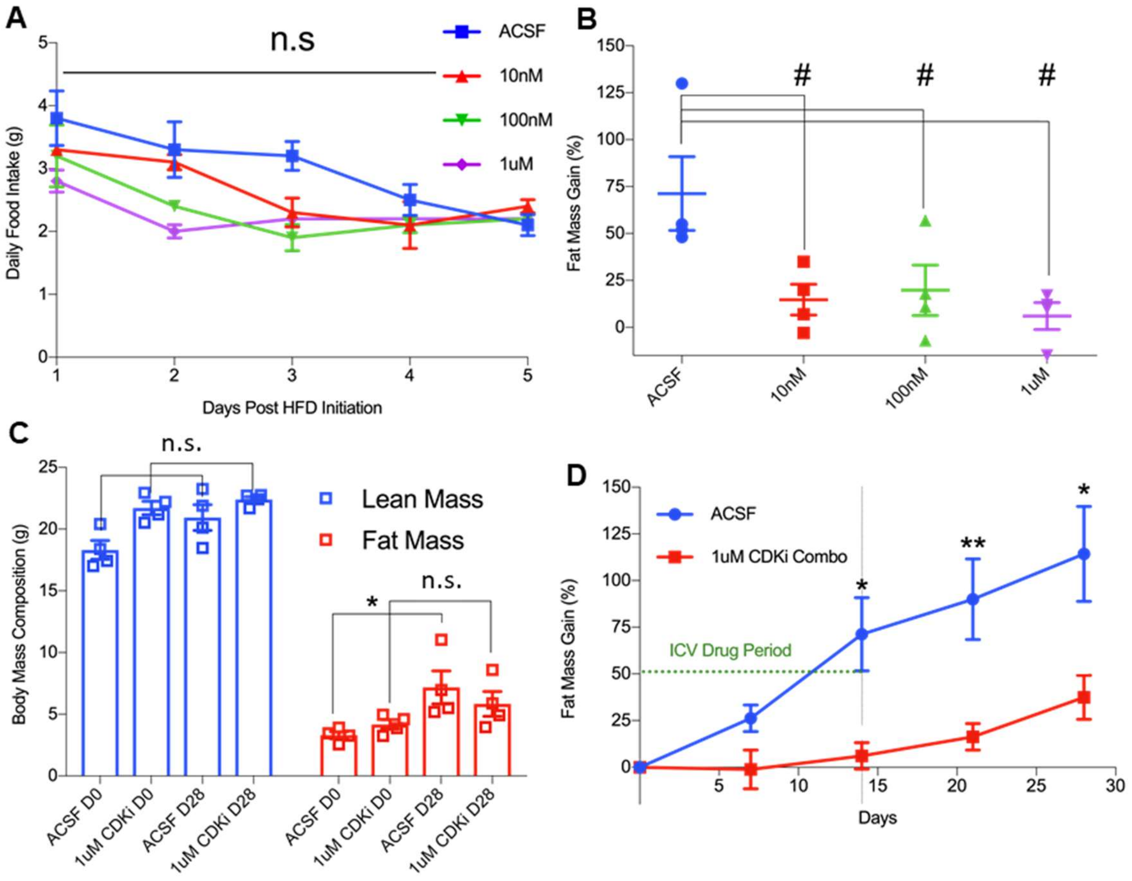

Supplemental Figure 4. ICV administration of abemaciclib and dinaciclib combination inhibits DIO. Two groups of weight and body composition matched male 6-week-old C57BL/6J mice were stereotaxically cannulated in the third ventricle. Daily injections for two weeks (Mon-Fri) consisted of 1 μL of abemaciclib-dinaciclib mix at the indicated concentration, or artificial CSF (ACSF) as control. HFD was initiated concurrently with the first injection. (**A**) Measurement of food intake during the first five days of injection. n = 4 for each dose group. (**B**) Fat-mass gain as percent of pre-HFD fat mass were measured at two weeks from injection start date. ACSF and 1uM abemaciclib and dinaciclib combination ICV cohorts were evaluated at two weeks following completion of ICV treatment. (**C**) Lean and fat-mass percent gain at the indicated days. (**D**) Fat mass gain (%) during the two week icv drug treatment period and two week post treatment period. Error bars represent SEM. *, $p < 0.05$, **, $p < 0.01$ by two tailed Student's *t* test. #, adjusted $p < 0.05$, by parametric one-way ANOVA with Bonferroni correction.

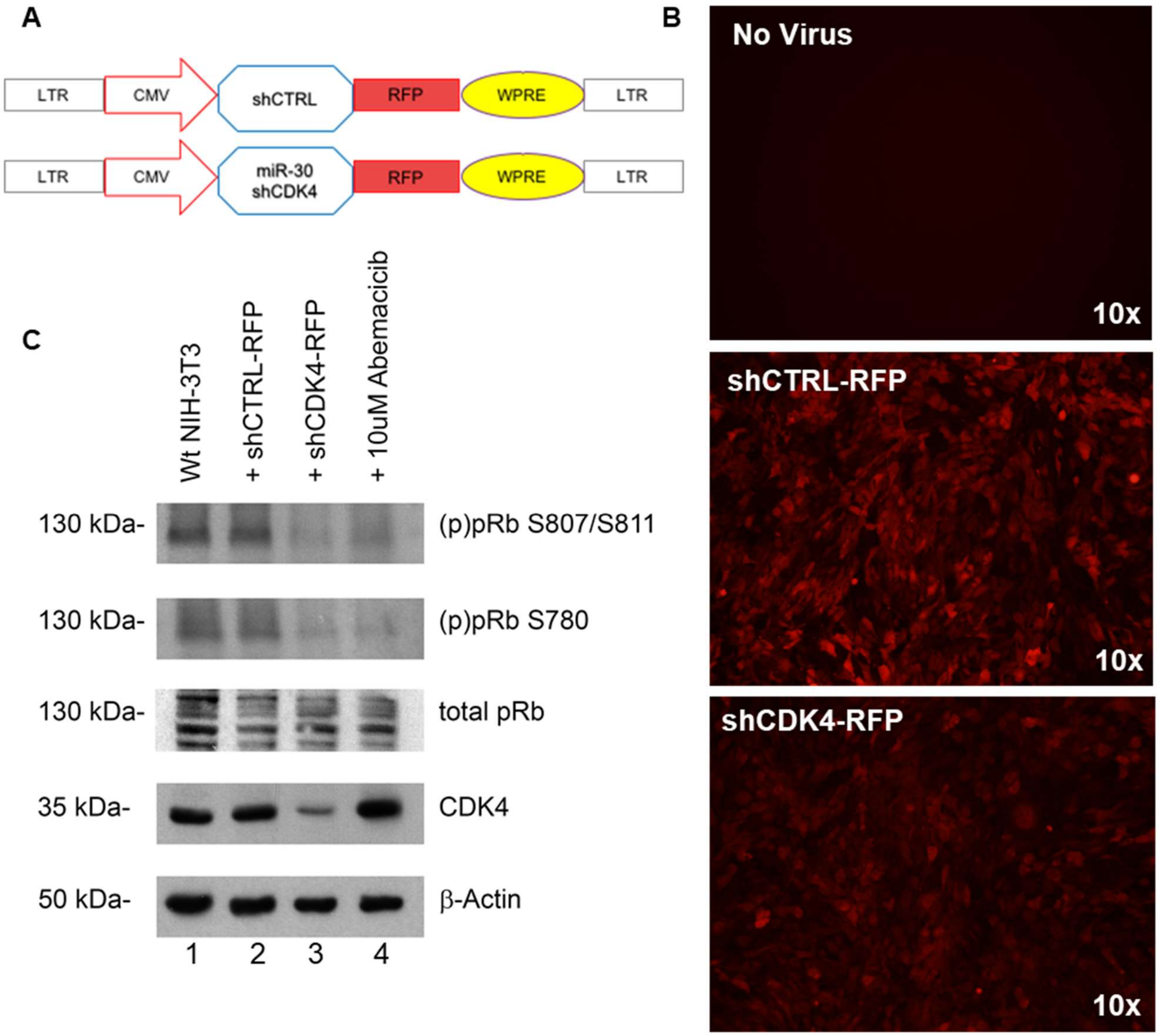

Supplemental Figure 5. CDK4 is required for hyper-phosphorylation of pRb. Exponentially growing NIH3T3 cells were transduced with (**A**) lenti-CMV-shCTRL-RFP, containing a 96 base-pair random scrambled nucleotide sequence immediately upstream of a Red Fluorescent Protein open reading frame (lane 2), or a 96 b.p. miR30 based mouse CDK4 knockdown shRNA sequence upstream of RFP (lane 3), or treated with 10 μM abemaciclib overnight (lane 4). (**B**) Native RFP showing transduction of NIH3T3 cell with the control and knockdown lentiviruses. miR-30 based knockdown involves microRNA processing of the hairpin and degradation of the mRNA, reducing RFP expression (*45*). (**C**) Cell lysates were prepared for Western blotting with pRbS807p/S811p and pRbS780p antibodies to determine the degree of pRb hyper-phosphorylation. Exponentially growing NIH3T3 cells (lane 1), lenti-CMV-shCTRL-RFP, containing a 96 base-pair random scrambled nucleotide sequence immediately upstream of a Red Fluorescent Protein open reading frame, transduced NIH3T3 cells (lane 2), lenti-CMV-miR30 based mouse CDK4 knockdown shRNA sequence upstream of RFP, transduced NIH3T3 cells (lane 3), or treated with 10 μM abemaciclib overnight (lane 4).

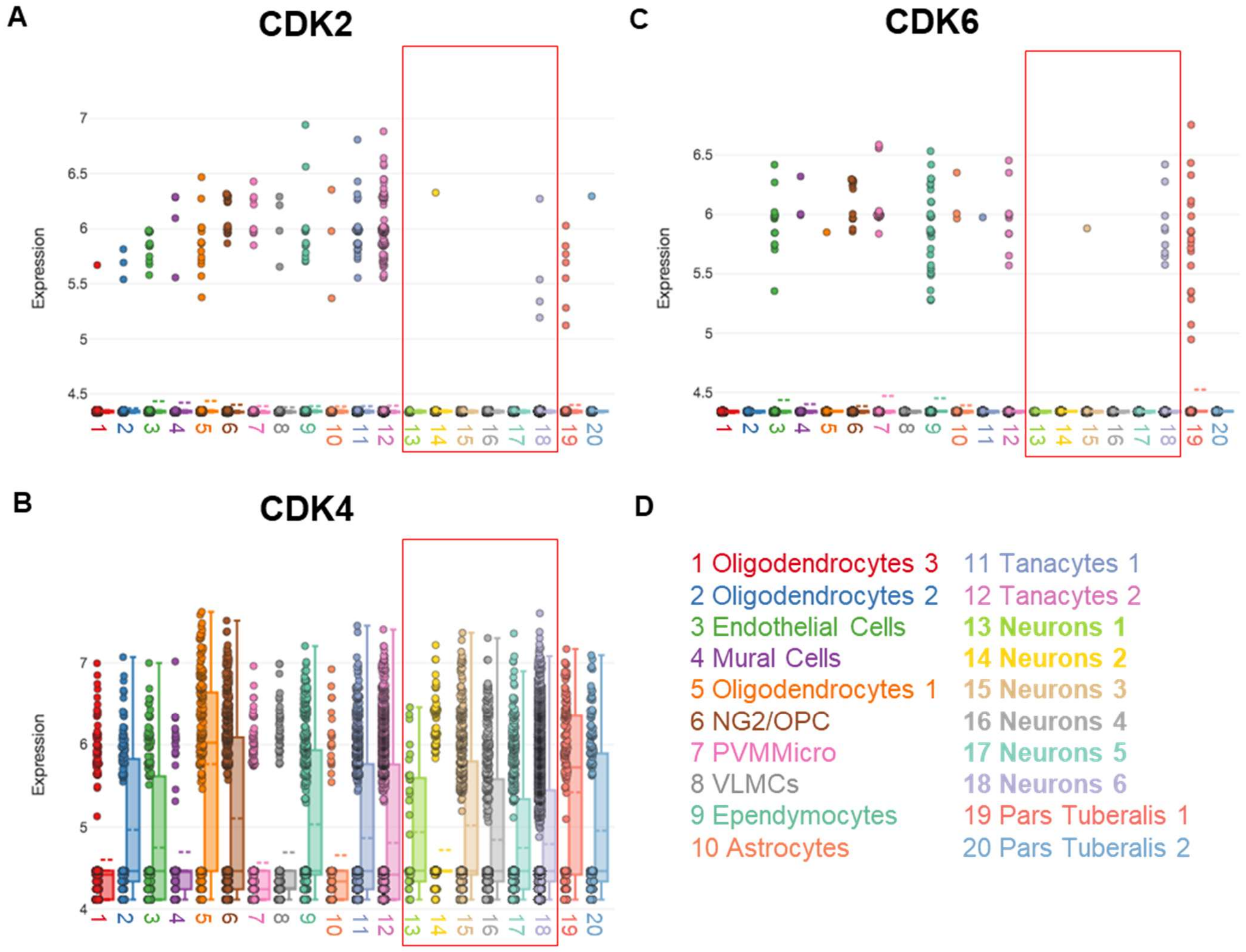

Supplemental Figure 6. Expression of pRb phosphorylating kinases in arcuate nucleus neurons. A molecular census of the arcuate nucleus (ARC) profiled gene expression in 20,921 individual cells in and around the adult mouse ARC using single cell droplet RNA-sequencing. Boxed plots summarize expression of CDK2 (**A**), CDK4 (**B**), or CDK6 (**C**) in specific cell-type clusters; POMC neurons are largely clustered within group 18. Each data point shows single neuron expression level of the indicated gene. Expression level is measured as the natural log of the number of unique molecular identifier (UMI) transcript counts per gene, normalized by 10,000 counts per cell. X-axis denotes specific cell-type clusters (**D**), determined using Seurat v2.1's FindMarkers function, and a cell-specific marker threshold set at 70 counts per transcript 10,000 counts. Neuron-specific clusters (13 through 18) highlighted inside red boxes. Data are presented using Broad Institute Single Cell Portal (https://portals.broadinstitute.org/single_cell). For full study details, please see *(27)*.

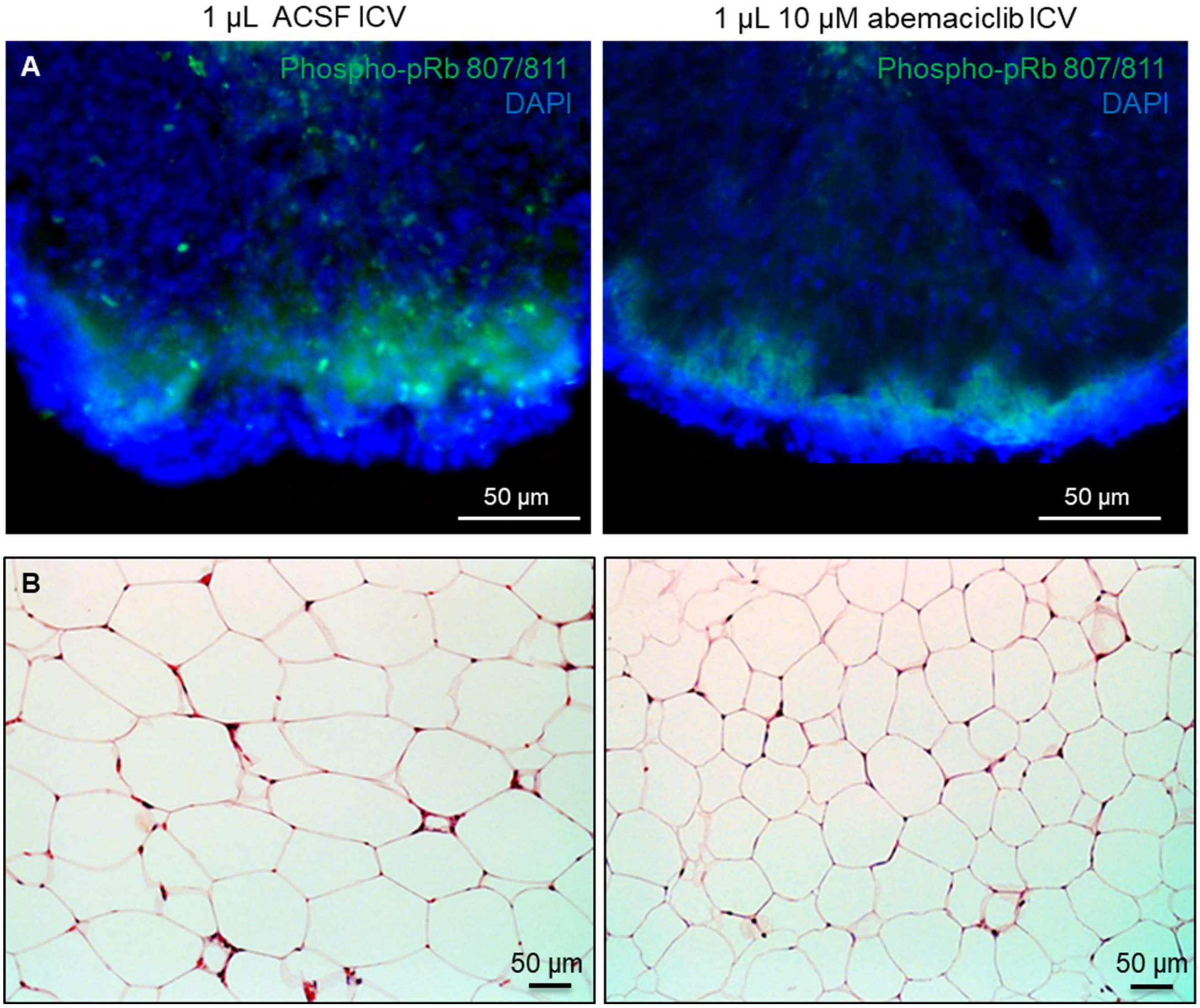

Supplemental Figure 7. ICV administration of CDK4/6 inhibitor abemaciclib inhibited pRb phosphorylation in the MBH and reduced adipocyte hypertrophy. Two groups of weight and body composition matched male 10-week-old C57BL/6J mice were stereotaxically cannulated in the third ventricle. Daily injections for two weeks (Mon-Fri) consisted of 1 µL of 10 µM abemaciclib or artificial CSF (ACSF) as control. HFD was initiated concurrently with the first injection. (**A**) Representative MBH sections from animal brains harvested upon conclusion of icv study are stained with pRbS807p/S811p antibody for phosphorylated pRb. (**B**) H&E stained gonadal fat pad section from ACSF treated mice, showing marked adipocyte hypertrophy and abemaciclib treated mice, showing reduced adipocyte hypertrophy. n = 5 per group.

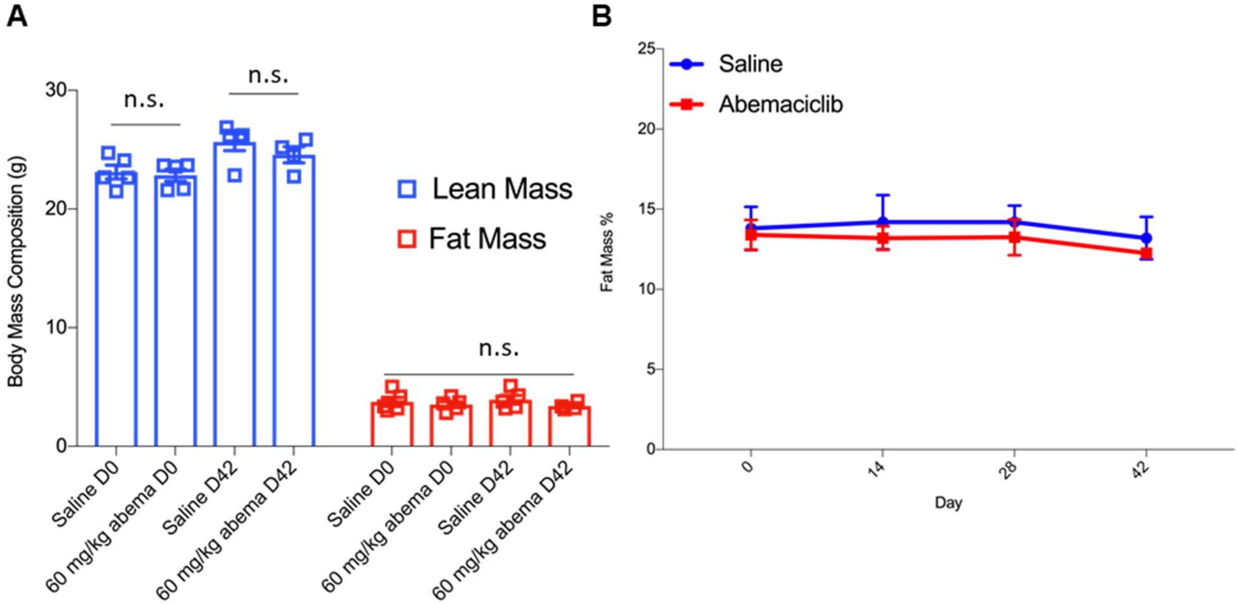

Supplemental Figure 8. Abemaciclib treatment has no effect on the body composition of normal chow diet fed mice. (**A**) Two groups of weight and body composition matched male 9-week-old C57BL/6J mice were continuously gavaged with abemaciclib (60mg/kg) daily for 42 days. Body mass compositions of mice after 42 days of treatment were assessed by MRI. (**B**) Fat-mass measured bi-weekly as percent of total body mass.

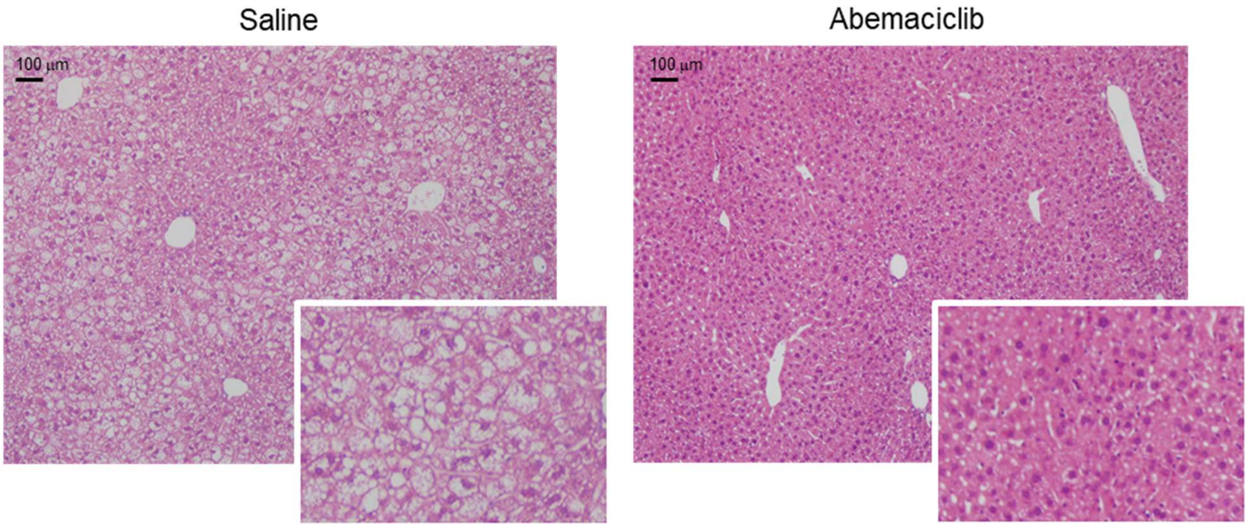

Supplemental Figure 9. Abemaciclib treatment reverses DIO-mediated heptaosteatosis. Two groups of weight and body composition matched male 9-week-old C57BL/6J mice were fed HFD for 4 weeks and matched into pairs based on similar body-mass composition for daily gavage with 60mg/kg abemaciclib or saline control concurrent with HFD. Shown are representative H&E stained liver sections after 21 days of treatment, showing reduced microvesicular fat accumulation in the hepatocytes of abemaciclib treated animals. scale bar = 100 μm. n = 6 per group.

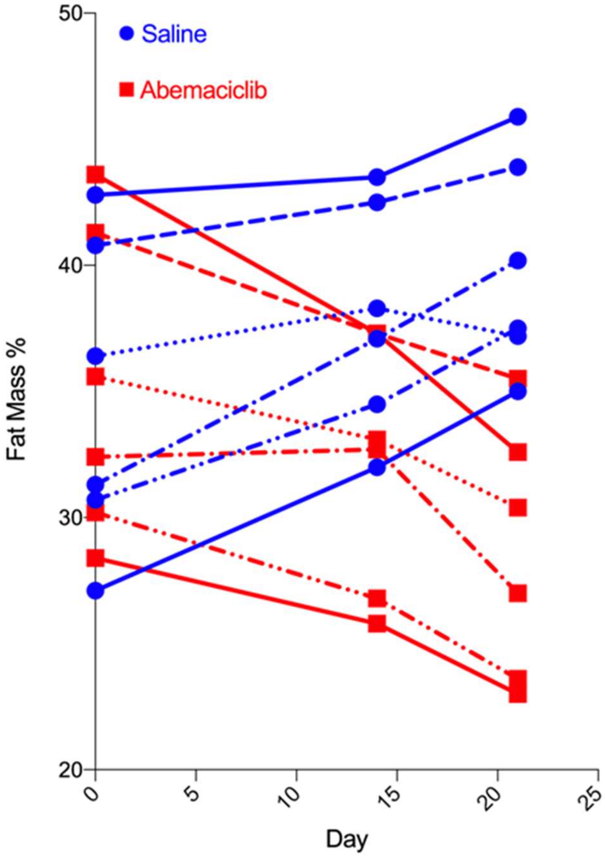

Supplemental Figure 10. Abemaciclib treatment reduces fat mass in DIO mice regardless of pre-treatment body composition. Two groups of weight and body composition matched male 9-week-old C57BL/6J mice were fed HFD for 4 weeks and matched into pairs based on similar body-mass composition for daily gavage with 60mg/kg abemaciclib or saline control concurrent with HFD. Body fat mass percents of individual animals were measured at indicated days during treatment. Similar line patterns indicate pre-treatment body-composition matched animal pairs. n = 6 per group.
